# Conformational changes underlying electromechanical transduction in prestin resemble a transport transition in pendrin

**DOI:** 10.64898/2026.06.06.730624

**Published:** 2026-06-08

**Authors:** Chenou Zhang, Richard Mariadasse, Jie Yang, Jun-Ping Bai, Joseph Santos-Sacchi, Dhasakumar Navaratnam, Oliver Beckstein

## Abstract

Prestin (SLC26A5), a membrane protein in cochlear outer hair cells, drives electromechanical transduction essential for mammalian hearing. Unlike other SLC26 anion transporters, prestin functions as a voltage-dependent molecular motor, transitioning between compact and expanded conformations. How this transition relates to the transporter cycle of SLC26 family members remains unclear. Here, multi-microsecond molecular dynamics simulations starting from the compact state reveal a rapid, spontaneous transition to an expanded state that resembles the inward-facing conformation of the anion exchanger pendrin (SLC26A4 from mouse). An accompanying transmembrane area expansion is localized to the inner membrane leaflet, likely leading to membrane bending. In line with this observation, reduced unitary sensor charge movement accompanies neutralization of charged residues localized near the inner leaflet. Simulations also uncover a previously uncharacterized compact conformation resembling outward-facing pendrin and predict an extracellular anion-binding site in prestin. In fact, in the presence of thiocyanate anions, we observe a previously unresolved binding site in a 3.27-Å cryo-electron microscopy structure of prestin. Furthermore, like prestin, pendrin exhibits a non-linear capacitance, an indication of voltage-dependent conformational switching. Together, these findings suggest that prestin and pendrin share core structural and functional properties, notably parallels between expansion-contraction states and transporter function, though transition speeds may differ.

## Full Text Availability

The license terms selected by the author(s) for this preprint version do not permit archiving in PMC. The full text is available from the preprint server.

